# Exposure to neonicotinoid pesticides induces physiological disorders and affects color performance and foraging behavior in goldfish

**DOI:** 10.14814/phy2.16138

**Published:** 2024-07-30

**Authors:** Izuru Kakuta, Kiyomi Takase

**Affiliations:** ^1^ Faculty of Science and Engineering Ishinomaki Senshu Univerisity Ishinomaki Miyagi Japan; ^2^ Research Center for Creative Partnerships Ishinomaki Senshu University Ishinomaki Miyagi Japan

**Keywords:** color performance, foraging behavior, goldfish, neonicotinoids, paddy field and laboratory test, physiological dysfunctions

## Abstract

We investigated the effects of neonicotinoid pesticides (NEOs) on the spontaneous swimming and foraging behavior, as well as the morphological and physiological changes of goldfish. Most fish reared in thiamethoxam (THM)‐sprayed rice fields showed the scales easily peeled off, and increased ascites. Some individuals showed decreased bio‐defense activity and low plasma Ca^2+^. Similar changes were found in the exposure test to THM (1.0 and 20.0 μg/L) and dinotefuran (1.2 and 23.5 μg/L). Next, the effects of a low concentration of THM (1.0 μg/L) on the spontaneous swimming and foraging behavior of fish were examined. Fish exposed to THM for 1 week became restless and had increased the swimming performance, especially under natural light, white LED lighting and blue LED lighting. Goldfish exposed to THM had also increased intake of shiny white beads under green LED illumination. These results indicate that the exposure to NEO, even for a short period and at low levels, not only suppressed bio‐defense activities and metabolic abnormalities, but also stress response, the swimming and foraging behavior of the fish are likely to be significantly suffered.

## 1 INTRODUCTION

Neonicotinoid pesticides (NEOs), developed in the 1990s, are considered to have insect‐selective toxicity and have been widely applied worldwide. However, it has recently been reported that NEO has a high possibility to induce dysfunction not only in pests, but also in beneficial insects such as honeybees and dragonflies of the red family, fish, and even mammals including humans (Aoda et al., 2013; Costas‐Ferreira & Faro, 2021; Gibbons et al., 2015; Tasman et al., 2021; Weili et al., 2015). In Europe, regulations have been tightened in accordance with the precautionary principle, but in Japan there has been very slow movement towards a ban on the use of these pesticides.

For fish, it has been reported that rearing zebrafish *Danio rerio* in water containing imidacloprid (300, 1250 and 5000 mg/L) for 21 days increases oxidative stress in the gastrointestinal tract (Weili et al., 2015) and exposure of African catfish *Clarias garipenus* to thiamethoxam (THM: 5 mg/L) increases blood alanine transaminase (ALT) and alkaline phosphatase (ALP) activity (Euony et al., 2020). It has also been reported that the exposure to imidacloprid (0.2–2000 μg/L) on zebrafish and Japanese medaka, *Oryzias latipes* (Vignet et al., 2019) and the exposure to thiacloprid (4.5–450 μg/L) on carp *Cyprinus carpio* embryos (Velisek & Stara, 2018) subsequently caused decreased survival, growth stagnation and behavioral abnormalities. Feminization has been reported to be induced in adult zebrafish exposed to acetamiprid (0.19–1637 μg/L) for 154 days. Acetamiprid and its metabolite were transferred to offspring, and they impaired to offspring production and development (Ma et al., 2022). When juveniles of the cyprinid fish *Alburnus alburnus* were exposed to 78 mg/L THM (using the insecticide Actara containing THM as active ingredient) significant increases in carbohydrate, lipid, and protein levels were observed after 28 days of treatment (Rania & Zaidi, 2023). Furthermore, it is reported that zebrafish exposed to a low concentration of THM (0.1 μg/L) for 45 days exhibited a range of abnormal behaviors, including anxiety, hyperactivity, high aggregation, excessive panic, and memory (Yang et al., 2023). However, there is still insufficient research on the effects of NEO on aquatic vertebrates, including issues such as what non‐NEOs would be better for animals and the environment if the use of NEOs were discontinued.

In Miyagi Prefecture, positive efforts have been made to promote organic and conservation agriculture since the mid‐1990s. In particular, the Tome region (located in the northern part of Miyagi Prefecture) has been an early promoter of the environmentally friendly rice movement, including the dissemination of cultivation methods that comply with ecological cultivation methods (Environmentally Friendly Rice Network Research Committee, 2006) (such as specially cultivated rice produced in accordance with the labelling guidelines for specially cultivated agricultural products and restrictions on the use of chemical pesticides that are stricter than the standards of the guidelines). In fact, over the past 20 years, the overall concentration of pesticides in rice paddies and surrounding waters has been declining considerably (Kakuta, 2004). However, significant reductions in the concentrations for certain pesticide components have not yet been recognized, and although efforts towards the total elimination of NEOs have started, the movement has been extremely slow and, even at present, THM is still detected in the water of nearby rivers, mainly in early summer, although it is below a few per cent of the environmental standard value (Iwata et al., 2022; Kakuta, 2004).

Among the NEOs, dinotefuran (DT), which is widely used mainly in early summer, is not sprayed in this area, and the concentration in paddy fields and surrounding waterways and rivers is always below the detection limit (Iwata et al., 2022). However, the pesticide is widely used in the prefecture and it is also the NEO with the largest amount of use in Japan. In many agricultural area in Japan, however, pesticide formulation containing DT continues to be used (General Incorporated Association Act beyond trust, 2016). Therefore, we investigated the effects of THM and DT on the physiology and behavior of fish. For comparison, it was also investigated the effects of exposure to fipronil (FN). Although FN is not a NEO, it is a highly penetrating insecticide and adverse effects on organisms, including fish, are of concern.

In this study, therefore, the effects of NEOs on goldfish were investigated by the following procedures: (1) the field test for 3 weeks in THM‐sprayed and non‐prayed rice fields, (2) the indoor test using pesticide formulations (THM, DT and FN) through tests using morphology and blood parameters as indicators, (3) the effects of a low level of active ingredient THM on spontaneous swimming speed and foraging behavior of fish.

## 2 MATERIALS AND METHODS

### 2.1 Fish

Goldfish (*Carassius auratus*) weighing 10–25 g purchased from commercial aquaculture companies, pre‐reared in dechlorinated tap water for at least 1 week were used for the research. Goldfish belonging to the Cyprinidae family have been bred in Japan for a long time as ornamentals and research subjects, and in addition to a lot of information on their behavior and physiology. Furthermore, this is because the goldfish used as the experimental fish can survive in the high temperature, low oxygen environment of rice fields.

### 2.2 Paddy fields for fish rearing trials

Six rice paddies in the Tome region of Miyagi Prefecture were selected as fish rearing sites: three are rice paddies without NEOs sprayed and the others with NEOs. The NEO applied as seedling box‐treated systemic insecticides in the area was thiamethoxam (THM): (EZ)‐3‐(2‐chloro‐1,3‐thiazol‐5‐yl ethyl)‐5‐methyl‐1,3,5‐oxadiazinan‐4‐ylidene(nitro)amine: product name; Digital Coratop Actara box granules or Digital MegaFlare box granules. Water samples for pesticide analysis were collected at the beginning of the exposure test of fish and at the time of picking up the fish (1 and 3 weeks after the start of exposure), using a polypropylene syringe with a capacity of 50 mL from the area around the container containing the fish and from the middle layer (several centimeters deep) of water collected near the outlet in the paddy field. And then, water samples filtered through a glass fiber filter with a pore diameter of 1 μm. Detection of THM in water was performed using liquid chromatography–tandem mass spectrometry (LC/MS) as described later. Each THM content was 2.0% or 8.0%. In the above rice paddies, rice planting took place from late May to early June, and THM was applied to paddy rice seedlings at the planting time.

### 2.3 Rearing trials in paddy field (outdoor exposure tests)

**FIGURE 1:**
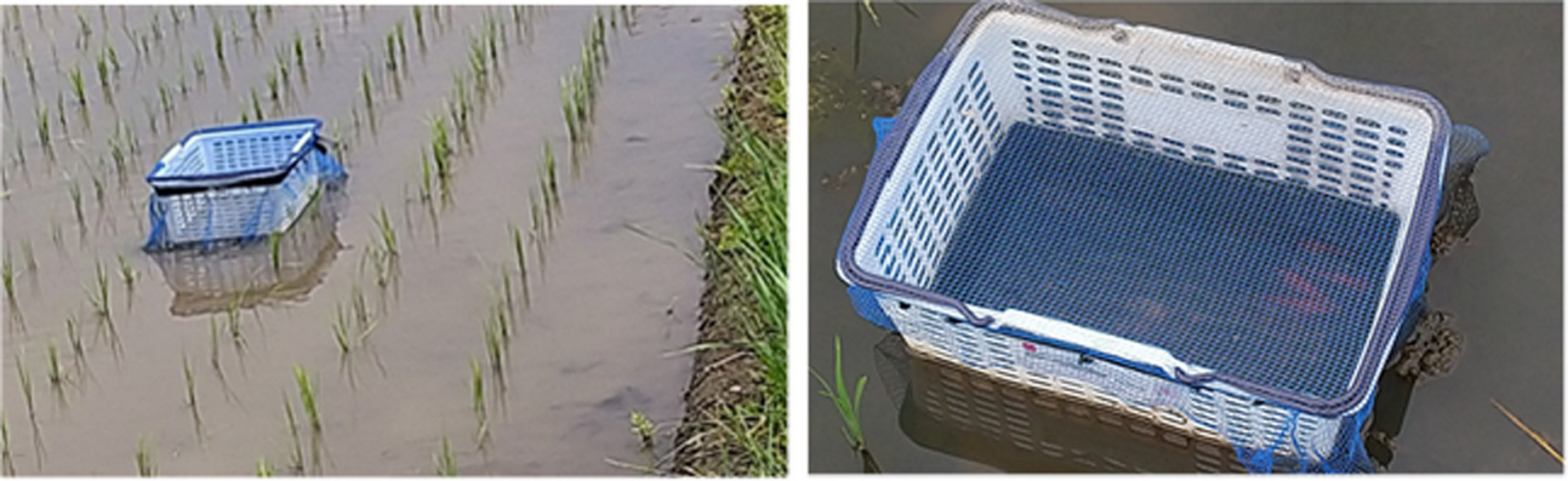
Rearing cages (shopping baskets with nets attached to the top, 510 × 240 × H 360 mm) placed close to the drainage outlet of the paddy field.

The outdoor exposure test was conducted from late May to June 2021, in line with rice planting. Rearing cages (shopping baskets with nets attached to the top, 510 × 240 × H 360 mm, Figure 1) were placed close to the drainage outlet in each of 3 paddy fields not sprayed with NEOs and 3 paddy fields sprayed with THM. In the study, 15 immature goldfish (weighing 10.0 ± 0.6 g; gonad weight less than 1% of body weight) were accommodated in each rearing cage. Five fish were picked up from each cage after 1 and 3 weeks of rearing and used for the following studies.

Blood samples were collected from the heart or the caudal vessels of a fish that has been stunned by a blow to the skull, using a syringe coated with sodium heparin. Individuals were also dissected after blood sampling to measure their weight and length, and to investigate the size and coloration of various visceral organs. The degree of obesity (condition factor) was determined from the following formula: body weight × 10^6^/(body length)^3^, body weight (g) and body length (mm).

Concentrations (μg/L) of THM in NEOs‐used paddy fields (*n* = 3) at the same study period were as follows: at the start of rearing (immediately after rice planting); 1.4 ± 1.0, 1 week later; 13.0 ± 3.2, 2 weeks later; 2.5 ± 1.9, 3 weeks later; 0.3 ± 0.2 and 4 weeks later; 0.1 (detection limit) or less. On the other hand, the concentrations in paddy fields where NEOs were not used were below the detection limit. For pesticides applied for registration before 3 August 2006, the paddy standard for THM is 500 μg/L, while the same standard for pesticides registered after that date is 47 μg/L (Minister of the Environment, Japan, Agricultural Chemical Registration Standards for Water Pollution, 2010).

In the paddy fields at the time of the study, there were no visible organisms in the early stages of exposure, regardless of whether the pesticide was used or not, but at the end of the exposure test (nearly 3 weeks later), some unidentified algae masses were drifting near the water surface.

### 2.4 Laboratory exposure studies using neonicotinoid active ingredients

#### 2.4.1 Effects of pesticide formulations on fish morphology and blood parameters

The NEOs THM (Thiamethoxam Reference Material, CAS RN®; 153719‐23‐4, Fujifilm Wako Pure Chemical Corporation) and dinotefuran (DT: (RS)‐1‐methyl‐2‐nitro‐3‐(tetrahydro‐3‐furylmethyl)guanidine, Dinotefuran Standard for Pesticide Residue Analysis, CAS RN®; 165252‐70‐0, Fujifilm Wako Pure Chemical Corporation), and a phenylpyrazole insecticide fipronil (FN: 5‐amino‐1‐[2,6‐dichloro‐4‐(trifluoromethyl)phenyl]‐4‐[(trifluoromethyl)sulfinyl]‐1H‐pyrazole‐3‐carbonitrile, Traceable Reference Material, CAS RN®; 120068‐37‐3, Fujifilm Wako Pure Chemical Corporation) which is widely used despite being a systemic insecticide with high biological impact (Ghaffar et al., 2018), were selected as pesticides to study effects on fish. In the laboratory exposure tests, commercial pesticide formulations were used in order to assess the effects on fish inhabiting the aquatic environment, including paddy fields. The names and active ingredient content of the used pesticide formulations containing the pesticide ingredients under investigation are as follows: THM; Actara granular water soluble 10%, DT; Alvarin granular water soluble 20%, FN; Prince granular 1%.

Goldfish were reared in breeding water containing dissolved pesticide formulations, including NEOs, for up to 4 weeks to determine the effects of the pesticides. The fish were divided into two groups, unsexed individuals whose gonads weighed less than 2% of their body weight and sexually mature individuals whose gonads weighed more than 4% of their body weight (12.8 ± 3.8 g and 16.8 ± 5.1 g, respectively). This procedure was performed to determine whether the effects of exposure to the pesticides differed by sexual maturation and by the sexes.

The rearing containers were glass aquaria with a capacity of 60 L, with a top filtering tank (only wool mats were used as filter media to remove suspended solids), and rearing tests were conducted under aerated conditions. In principle, 20 fish were kept in each tank. Fish were maintained on a 10L:14D (10‐hour‐light:14‐hour‐dark) photoperiod at 20°C. During the exposure test, 1% of the fish weight was administered every other day, and the fish were cleaned of residual food and feces 3 h after feeding.

The concentrations (μg/L) of THM, DF and FN in this exposure test are 1.0, 1.2, and 0.01 for low (L) and 20.0, 23.5, and 0.2 for high (H) concentrations, respectively. Approximately two‐thirds of the rearing water was replaced every 3 days, and just to be sure, the water collected from each tank almost every week was filtered through a glass fiber filter with a pore size of 1 μm, and THM and DF were detected by LC–MS/MS, and FN was detected by GC/MS. Quantitative analysis of each pesticide component was conducted by Nippon Steel Eco‐Tech Co. and Shokukanken Inc.

Ministry of the Environment's pesticide registration standards (reference values) for each pesticide component in relation to the prevention of damage to living environment animals and plants in water bodies are 3.5, 12, and 0.024 μg/L, respectively (Minister of the Environment, 2016, 2017).

Fish taken up 1, 2, and 4 weeks after the start of the exposure test were examined for the external appearance, and the weight and the conditions of internal organs. Blood was also collected from the heart or tail vessel of goldfish using a heparinized syringe, and the biodefence activity and the health indices in the blood were measured.

#### 2.4.2 Measurement of physiological indices

The number of red blood cell (RBC: 10^6^ units/μL) were determined by the blood cell calculator method after dilution of whole blood with 0.75% NaCl (Sodium Chloride Guaranteed Reagent, CAS RN®; 7647‐14‐5, Fujifilm Wako Pure Chemical Corporation). The blood was smeared on a glass slide, stained with May‐Grunwald's Eosin Methylene Blue Solution (E. Merck, Serial Number; 1.01424, Vendor; Fujifilm Wako Pure Chemical Corporation), and examined to measure the ratio of granulocytes and lymphocytes per RBC, and then the actual number of each blood cell was calculated using the number of RBC measured separately.

Saline suspended with 0.6 mg/mL of zymosan A from *Saccharomyces cerevisiae* (Sigma‐Aldrich Japan, CAS No. 58856‐93‐2) was mixed with whole blood in equal volumes, and the mixture was allowed to react for 30 min at 25°C with inversion and mixing every 5 min. The ratio of zymosan‐incorporated granulocytes per 100 granulocytes was determined as phagocytic activity (%). Potential killing activity (bactericidal activity) was also determined according to the usual method (Miyazaki, 1998).

After investigation of the above items, the blood was centrifuged (1200*g*, 10 min) to obtain a plasma fraction and the following items in the same sample were measured: ALT, Detaminer II (Manufacturing and sales notification number; 13A2X00172054003, Hitachi Chemical Diagnostics Systems, Company name changed to Minaris Medical Co., Ltd.) for automatic analyzer for alamine aminotransferase kit; ALP, Lab Assay TMALP (merchandise number; 633‐51021, Fujifilm Wako Pure Chemical Corporation), total antioxidant activity; Antioxidant Assay Kit (Item No. 709001, Cayman Chemical Company), and calcium (Ca^2+^); Metallo‐assay Calcium measurement LS (CPZIII) kit (Product code; CA01M, MG Metallogenics Co., Ltd.). Cortisol (a major stress hormone) levels in blood were also measured using the Cortisol EIA Kit (merchandise number; EA65, Oxford Biomedical Research Inc.).

#### 2.4.3 Effects of THM (active ingredient) on fish spontaneous swimming and foraging behavior

Fish exposed to a 1 μg/L THM solution for 1 week were transferred to each 300 mm diameter circular aquarium containing 5 L of fresh water and were rested for 1 day. After fish were kept under natural light or light of various tones (white, red; a peak wavelength of 583 nm, green; a peak wavelength of 502 nm, blue; a peak wavelength of 433 nm LED light irradiation, and poorly lighted conditions) for 30 min, air supply was stopped, and spontaneous swimming behavior was examined for 30 min under the same light conditions. Fish behavior was recorded using a video camera (JVC GZ‐RX500‐B). The water surface illuminance (lux) of natural light, and white, red, green and blue LED lights was 157, 161, 134, 180, and 162, respectively. The brightness of the poorly lighted space was less than 10 lux. Then, spherical plastic beads (TOHO Queen Beads Round Small Beads, OKADAYA Co., Ltd.) of various colors (red; merchandise number 4964291071889, black; 4964291070233, yellow; 4964291071858, green; 4964291071902, blue; 4964291071872, white; 4964291070387, shiny white; 4964291073074) with a diameter of 2.0–2.2 mm, five each, were thrown into the aquarium containing fish that have been fasted for 1 day, and the number of beads put in the mouth within 5 min after throwing the beads was counted.

### 2.5 Statistics

The numbers in the text are expressed as mean ± standard deviation unless otherwise stated. The JMP 6 software (SAS Institute Japan Co., Ltd) was used to process and analyze the experimental data. Tukey's multiple range test after confirming equal variances was used multiple comparisons based on one‐ or two‐way ANOVA with *p* < 0.05 as the limit of significance.

## 3 RESULTS

### 3.1 Rearing trials in paddy field (outdoor exposure tests)

No individuals died in the exposure test for 3 weeks in the paddy field. On week 3, no difference in body weight was observed between the two types of paddy fields (fish weight was 11.9 ± 1.7 *g* in the pesticide free paddies and 11.8 ± 2.1 g in the THM‐used paddies).

**FIGURE 2:**
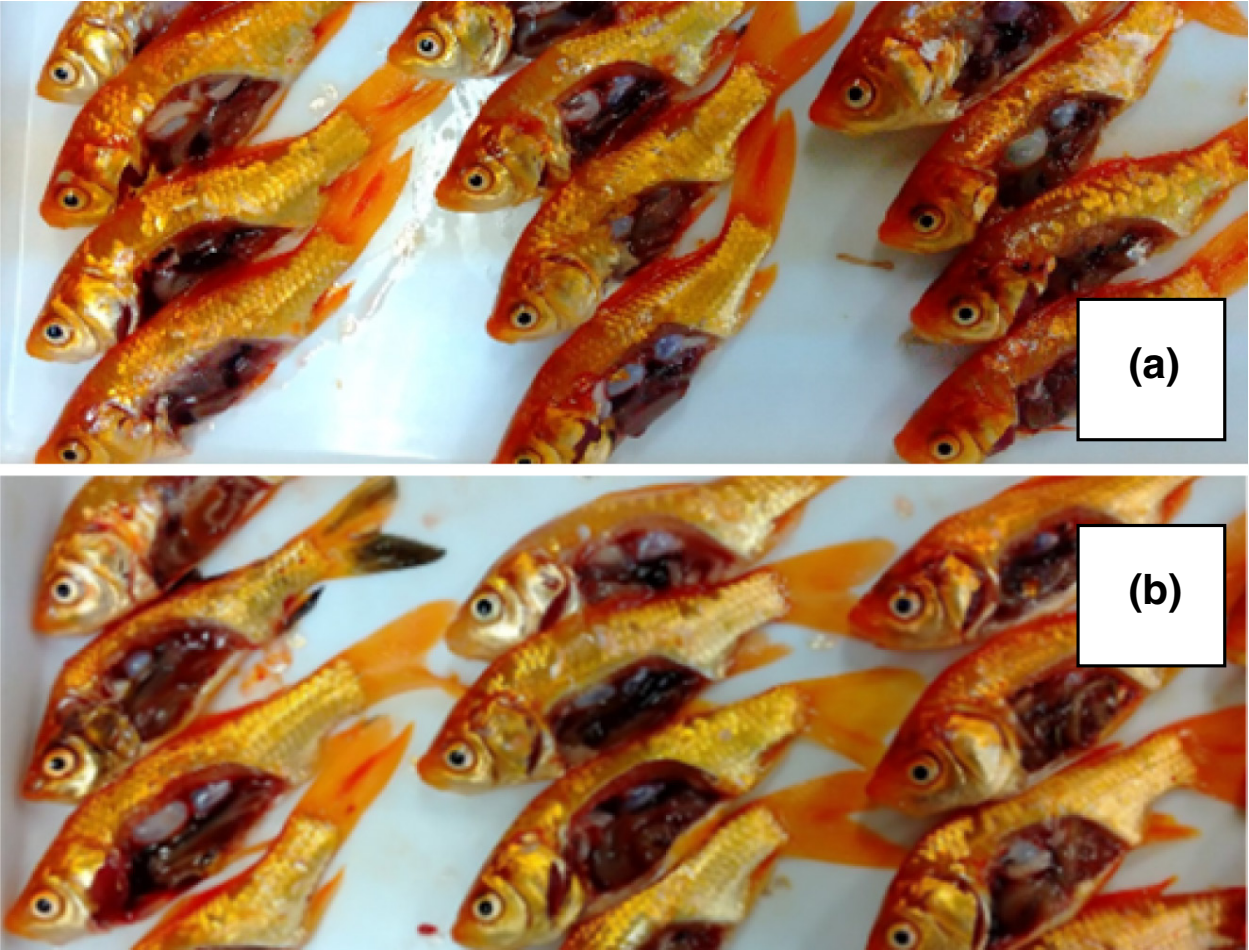
(a) Fish reared for 3 weeks in a cage set up in a paddy field without pesticides. (b) Fish reared for 3 weeks in a cage set up in a paddy field treated with a neonicotinoid pesticide (thiamethoxam). Fish reared for 3 weeks in the paddy fields sprayed with thiamethoxam had scales that were easily peeled off, and large amounts of ascites were often collected in the abdominal cavity. Blackening of the body surface was also observed in some individuals. Regarding internal organs, changes in the color of the liver, such as a milky white color due to excessive accumulation of lipid and green color due to cholestasis, swelling of the spleen, and decreased flexibility of the intestinal tract (fragile gastrointestinal tract) were observed.

The external appearance and the condition of the internal organs of the goldfish taken up after the three‐week rearing trial (mid‐June, before noon, cloudy, water temperature 28°C, pH 7.6) were as follows: Fish reared in THM free paddy field had firm abdominal skin, lateral muscles and internal organs, with no or slight presence of ascites in the abdominal cavity. One week after the start of rearing in the paddy field sprayed THM, the abnormal changes were hardly observed. On week 3, the fish in the paddy field sprayed THM had thin abdominal skin and muscle (thinning), scales that were easily peeled off, and often had a large amount of ascites fluid in the abdominal cavity. Body surface blackening was also observed in some individuals. Regarding the internal organs, changes in liver coloration such as milky white coloration due to excessive fat accumulation and greening due to cholestatic retention, swelling of spleen and reduction flexibility of the intestinal tract (fragile digestive tract) were observed in fish reared in paddy fields where THM was used (Figure 2).

In addition, at the end of the exposure tests in the experimental paddy fields (close to 3 weeks later), regardless of whether pesticides were used or not, some algae (unidentified) clumps were found floating near the water surface. Some of the fish taken from such paddies had black algal mass‐like objects accumulating in their digestive tracts. However, when comparing fish from each paddy field with and without algae‐like deposits in the digestive tract, no remarkable differences were observed in the appearance, condition of the internal organs and the following blood properties.

**FIGURE 3:**
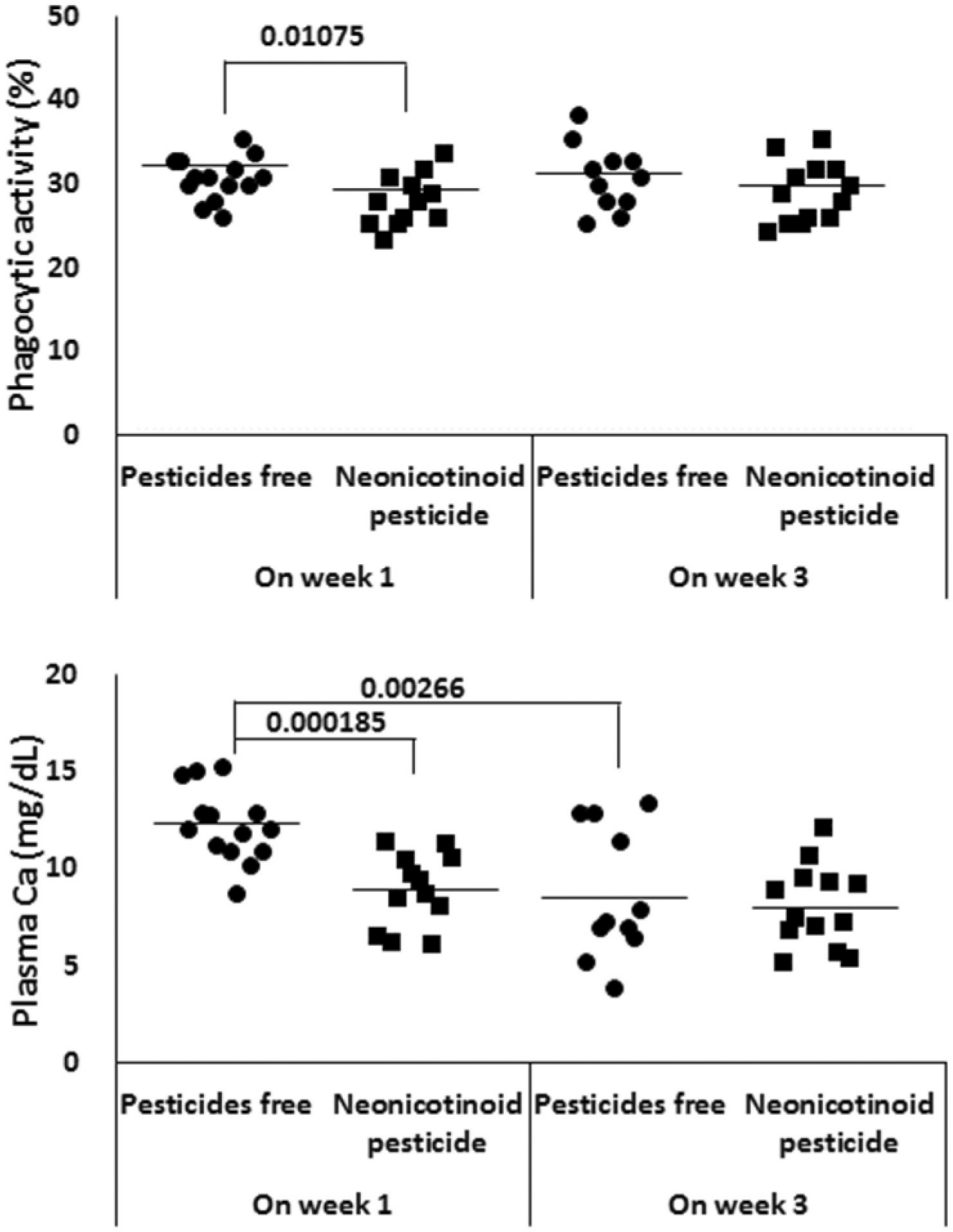
Changes in the condition factor (upper left), the phagocytic activity of granulocytes (upper right) and the plasma calcium concentration (lower) of goldfish in paddy fields without and with NEO (thiamethoxam). Each value from 11 to 14 fish per group and mean value. *p*‐value are by one‐way ANOVA with Tukey's post‐test for multiple comparisons.

Figure 3 shows the changes in the indices for which statistically significant differences were found depending on whether THM was used or not. Granulocyte phagocytosis activity and plasma calcium concentration were significantly lower in the pesticide‐used paddy fields (Figure 3 left). Plasma calcium concentrations of fish reared in pesticide‐free rice paddies for 3 weeks were significantly lower than those after the first week (Figure 3 right). Though a mean value from individuals reared in THM‐used paddy fields was more than three times higher than that of fish reared in non‐pesticide paddy fields, a significant change wasn't found in plasma ALT activity for a large coefficient of variation in it (0.84). Plasma cortisol levels, antioxidant capacity and ALP activity were also highly variable between individuals and at different times of fish sampling, and no significant differences were found between paddies.

### 3.2 Effects of exposure to pesticide formulations on fish morphology and blood parameters through laboratory exposure tests

In immature fish, no individuals died during the 4‐week exposure test in the low concentration groups of THM and DT, while in the high concentration group, fish died after 20 and 12 days, respectively. Fish died after 18 days in the low FN concentration group and after 9 days in the high FN concentration group. The percentage of fish dying after 4 weeks of pesticide exposure in the low and high concentration groups of THM, DT and FN were 0% and 10%, 0% and 15% and 5% and 20%, respectively. In contrast, in sexually mature fish, the death rates after 4 weeks of exposure to low and high concentrations of THM, DT and FN were 0% and 15%, 5% and 15%, and 10% and 30%, respectively. There was a slight trend towards increased mortality in sexually mature fish. In fish exposed to the above pesticides, regardless of sexual maturity, there was a tendency for the high DT concentration group and the low and high FN concentration groups to feed less and to move slightly more slowly in the second half of the test.

**FIGURE 4:**
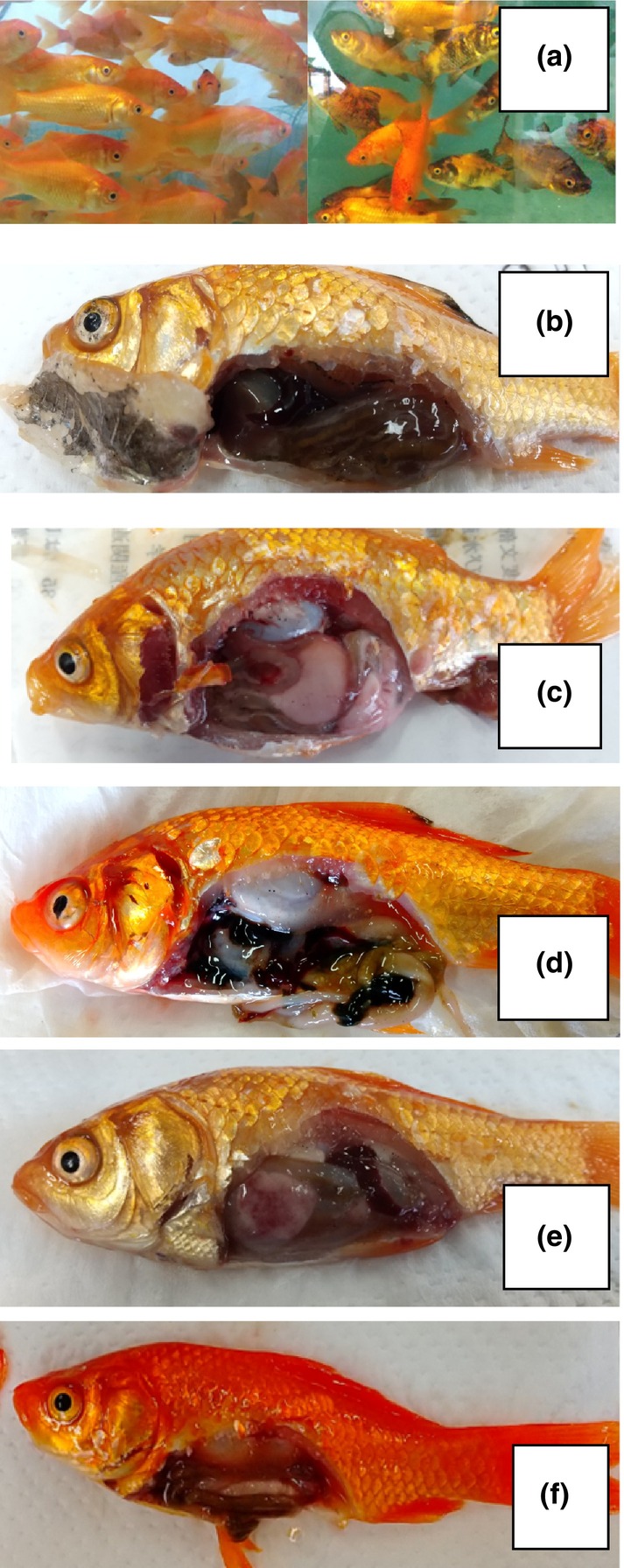
Macroscopic observation of external appearance and internal organs of goldfish exposed to neonicotinoid pesticides for 4 weeks. (a) Fish from the control group (left) and fish exposed to high concentrations (20 μg/L) of thiamethoxam (right). (b–d) Fish exposed to 20 μg/L thiamethoxam exhibit (b) blackening of the abdominal wall and greening of the liver, (c) opalescence of the liver due to enlargement and excess lipid accumulation, and (d) intestine with decreased elasticity. (e) Fish exposed to 23.5 μg/L dinotefuran exhibit opalescence of the liver due to lipid accumulation. (f) Fish exposed to 0.2 μg/L fipronil exhibit large amounts of ascites and swollen intestines with decreased elasticity.

Figure 4 shows the external appearance and macroscopic findings of internal organs of fish exposed to pesticides. In the group exposed to high concentrations of THM for 2 weeks, a phenomenon of partial darkening of the body surface (hypermelanosis) was observed. Detachment and thinning of scales, increased spleen specific index (spleen‐to‐body weight ratio), intestinal swelling with decreased elasticity, and a large amount of storage of ascites were observed in both low and high THM groups from 2 weeks onwards. In some individuals from the high‐concentration group, enlarged liver, milky coloration due to excessive fat accumulation (with a greater tendency to spread from the middle and late stages), and green coloration of the liver were also observed. In the DT‐exposed group, changes similar to those in the THM‐exposed group were observed, except for a decrease in the specific spleen index after 2 weeks. After 1 week of exposure to FN, 40% of the fish in the high‐concentration group showed green discoloration in the liver. A large of ascites retention and intestinal swelling with decreased elasticity were observed on 2 weeks and later, but the degree of abnormality was higher in the high‐concentration group.

No effect of exposure to THM, DT and FN was observed on the number of RBC (mean values of 1.39–1.71 × 10^6^ cells/μL), granulocytes (0.6–1.1 × 10^4^ cells/μL) and lymphocytes (0.7–1.2 × 10^4^ cells/μL) in immature goldfish. Sexually mature individuals also showed no significant changes in their respective blood cell counts.

**FIGURE 5:**
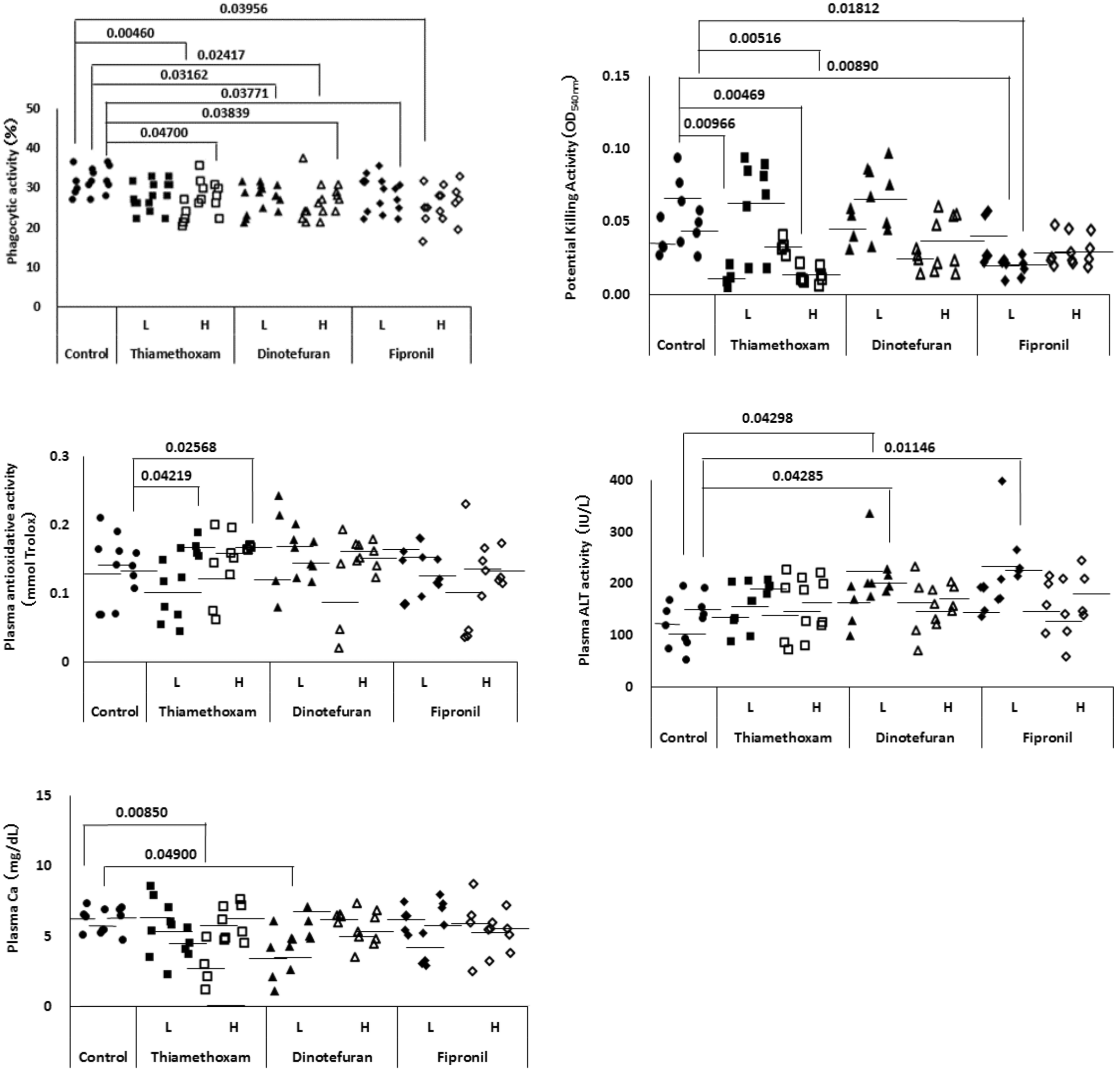
Changes in physiological parameters of immature goldfish experimentally exposed to low and high concentrations of thiamethoxam (L; 1 μg/L, H; 20 μg/L), dinotefuran (L; 1.2 μg/L, H; 23.5 μg/L) and fipronil (L; 0.01 μg/L, H; 0.2 μg/L). Upper left; granulocyte phagocytic activity, upper right; PK activity, middle left; plasma antioxidant capacity, middle right; plasma ALT activity, bottom left; plasma calcium concentration. Each value from five for phagocytic activity or four for other parameters fish per group and mean value. *p*‐value are by one‐way ANOVA with Tukey's post‐test for multiple comparisons. For each group, the results of the 1‐, 2‐ 1 and 4‐week exposure tests were shown from left to right.

Figure 5 shows the effects of exposure to THM, DT and FN on the phagocytic activity and PK activity of granulocytes and various physiological indices of plasma in immature goldfish. The phagocytic activity of granulocytes (Figure 5 upper left) was significantly lower in the high THM group after 1 and 4 weeks, in the low DT group after 4 weeks and the high DT group after 2 and 4 weeks, respectively. For FN exposure, significant low values were observed in the high‐dose group after 2 weeks and the low‐dose group after 4 weeks. Potential killing (PK) activity (Figure 5 upper right) was significantly lower in the high THM group after 2 and 4 weeks, and in the low and high FN groups after 2 weeks, respectively. Plasma antioxidant capacity (Figure 5 middle left) showed significantly higher values in the low and high concentration groups after 4 weeks of exposure to THM. For plasma ALT activity (Figure 5 middle right), significantly higher values were found in the low DT group after 2 and 4 weeks and in the low FN group after 4 weeks. For plasma calcium levels (Figure 5 bottom left), a significantly lower value was found only after 1 week in the THM and 2 weeks after in the DT low‐concentration group.

No effect of these pesticides was observed on plasma ALP activity. Mean plasma cortisol levels in the high THM and FN groups were remarkably higher on week 4. Due to the large individual differences in these groups, no statistically significant changes were observed in the experimental groups.

A similar exposure study in sexually mature goldfish showed slightly greater magnitude of change for most of the indices examined. In particular, plasma cortisol levels were significantly higher after 4 weeks in the high DT group. No sex differences were observed in the exposure test results.

### 3.3 Effects of THM (active ingredient) on fish spontaneous swimming and foraging behavior

**FIGURE 6:**
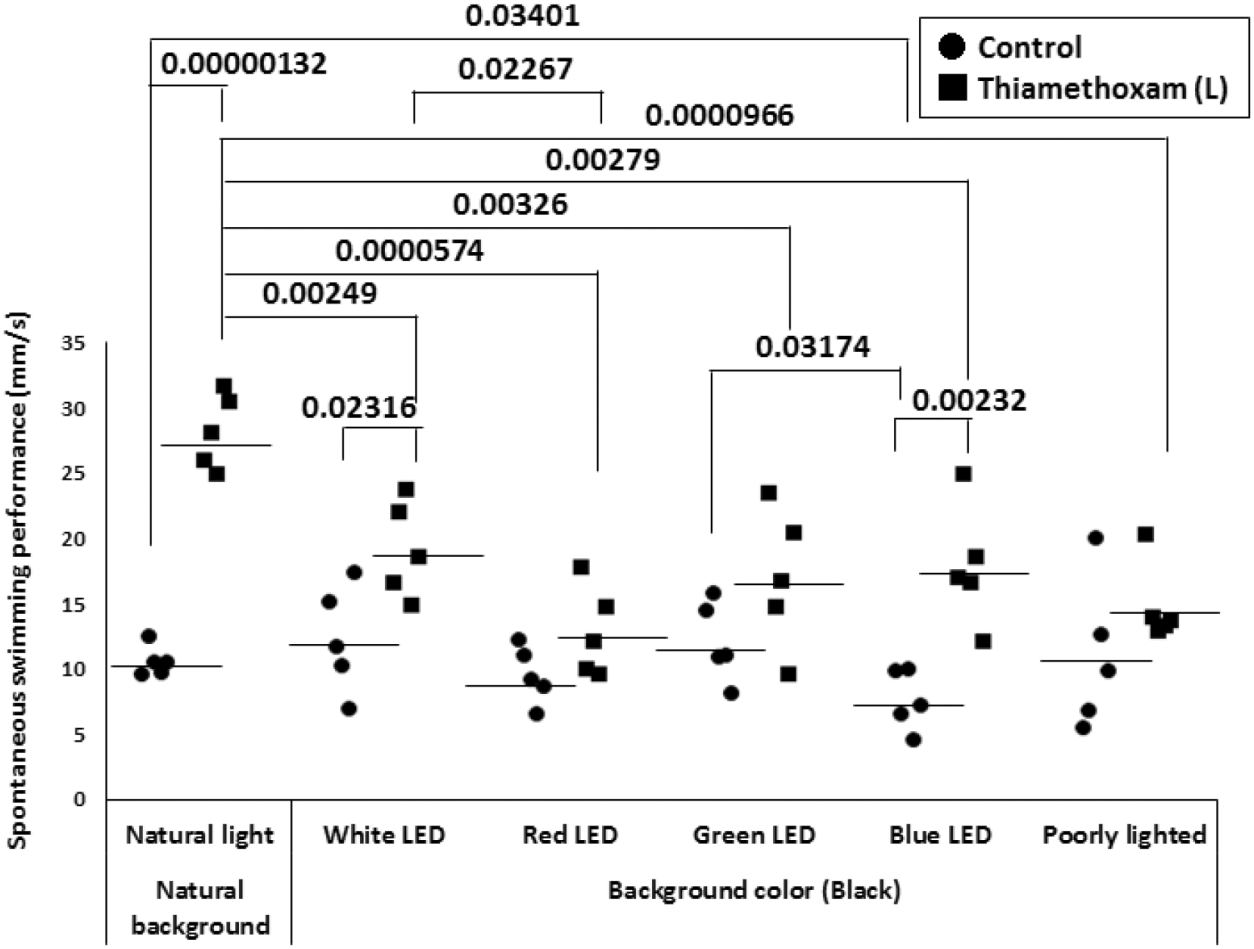
Effects of differences in light quality on spontaneous swimming performance of goldfish experimentally exposed to low concentrations of thiamethoxam (1 μg/L). Each value from five fish per group and mean value. *p*‐value are by two‐way ANOVA with Tukey's post‐test for multiple comparisons.

Figure 6 shows the effects of differences in light quality on spontaneous swimming performance of goldfish experimentally exposed to low concentrations of THM (1 μg/L). Differences in light quality did little significantly change the swimming speed of fish in the control group (under the blue LED, lower values were observed compared to natural light conditions). On the other hand, fish exposed to low concentrations of THM for 1 week showed a significant increase under natural light, white LED lighting, and blue LED lighting. Swimming behavior in fish exposed to thiamethoxam increased under all light conditions. There was no difference in the swimming speed between the THM‐exposed and control groups when tested under red LED lighting, green LED lighting, and poor lighted conditions.

**FIGURE 7:**
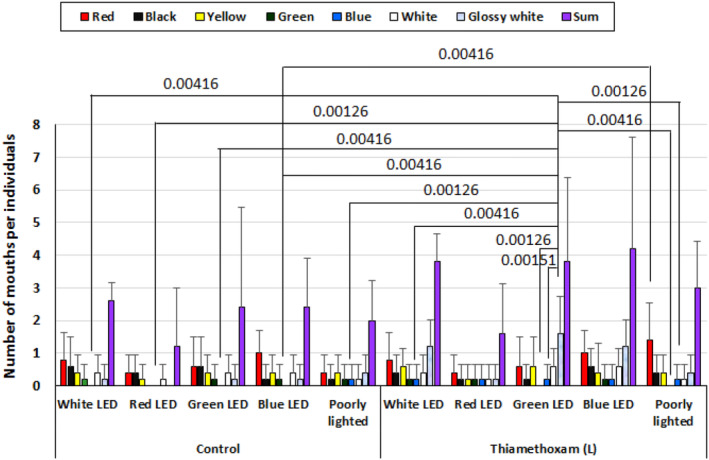
Effects of differences in light quality on the number of beads taken into the mouth per each goldfish exposed to low concentrations of thiamethoxam (1 μg/L). Mean ± standard deviation, number of individuals 5. *p*‐value are by two‐way ANOVA with Tukey's post‐test for multiple comparisons.

Figure 7 shows the effects of light quality and diet color on the feeding behavior of goldfish experimentally exposed to 1 μg/L THM solution for 1 week. Interactions (effect modifications) were observed between the presence or absence of THM exposure, differences in LED light color, differences in bead color, and between THM exposure and bead color. On the other hand, no interaction was observed between THM treatment and LED light color, or between LED light color and bead color. When investigating whether there were significant differences between the indices for which interactions were observed, significant differences were found between the frequency of ingestion of glossy white beads under green LED illumination in the THM‐treated group and the frequency of ingestion of blue beads when irradiated to a white LED, red LED, green LED, blue LED, and under poorly lighted conditions in the control group, and the frequency of intake of blue beads when irradiated to a white LED, green and blue beads when illuminated to a green LED, and green and blue beads under poorly light conditions in the THM‐treated group (the THM‐exposed group ingested more glossy white beads when exposed to a green LED). In addition, the frequency with which THM‐treated individuals ingested red beads under poorly lighted conditions was significantly higher than the frequency with which THM‐untreated individuals ingested blue beads under blue LED irradiation.

## 4 DISCUSSION

Goldfish reared in paddy fields sprayed THM for 1 week showed reduced phagocytic activity of granulocytes and plasma calcium levels compared to fish reared in paddy fields not sprayed with the same pesticide. On week 3, though these changes disappeared, some abnormalities such as thinning of the abdominal wall and tendency of scales to peel off, color changes due to enlargement of the liver and excessive accumulation of lipid, enlargement of the spleen, accumulation of ascites fluid, and intestinal swelling and thickening were observed in the goldfish exposed to THM. These changes showed that the fish reared in THM‐splayed paddy fields suffered from various physiological dysfunctions, not only suppressed biodefence activity, but also abnormallties in lipid, salt, and water metabolisms.

The reason why the plasma Ca concentration in the fish reared in THM‐splayed paddy fields was low only after 1 week is unknown, but after 3 weeks, while the same concentration recovered, thinning and exfoliation of the scales were observed. Therefore, there is no doubt that exposure to THM induced the abnormality in Ca metabolism of fish. Decreased extracellular blood calcium increases the irritability of nervous tissue, and its very low levels can cause spontaneous discharge of nerve impulses, leading to decreased brain activity, abnormalities in nerve transmission and muscle contraction (Hays & Swenson, 1985; Murray et al., 2000). Therefore, the decrease in plasma Ca concentration found in fish exposed to THM may be significantly related to changes in fish behavior and physiological mechanisms as described below, which is important for understanding the biological effects of exposure to NEO.

The concentration of THM in paddy field water after 1 week of exposure was approximately 4 times the Ministry of the Environment's registration standard for pesticides for the prevention of aquatic damage (Iwata et al., 2022), but after 2 and 3 weeks it was 0.7 and 0.1 times the standard, respectively. However, the physiological damage to the fish was more pronounced after 3 weeks, when the pesticide concentration of THM in the water was low and little significant changes in blood properties were observed. Our results show that damage caused by NEOs, at least THM and DT, cannot be easily improved even if the concentration of pesticides in water is reduced, and also suggest that it is extremely difficult to grasp the effects of neonicotinoid‐based insecticides for animals from blood properties, though many research on the changes of blood properties have been made to understand the effects of NEOs (Azadikhah et al., 2023; Bojarski & Witeska, 2020; Euony et al., 2020; Hong et al., 2018).

In most paddy field exposure trials, at the end of the study (nearly 3 weeks later), algal masses were seen drifting near the water surface. Some fish reared in such paddy fields had similar objects lodged in their digestive tracts on week 3. Freshwater fish basically do only a little drink water (Kobayashi et al., 1983), but it is known that they drink water under stressed conditions (Evans, 1993), so THM enters fish body through the digestive tract, as well as the gills and skin.

Against the concentration of THM in the water of paddy fields sprayed pesticides decreased from one‐fifth (after 2 weeks) of the value on week 1 to one‐twentieth (after 3 weeks) of that, the pesticide contents increased in algae and small animals living in it (food source) by means of biomagnification and bioaccumulation. Therefore, it may be that around the end of the study more pesticides entered the body via the gastrointestinal tract than via the gills and the skin from the water source. However, it cannot be denied that the exposure tests in paddy fields did not provide sufficient information on contamination routes, as the pesticide content within the algae was not measured in this study.

Furthermore, as the same type of pesticide is actually used intermittently over a wide area, the period of high concentrations of the specific pesticide may last longer for aquatic animals, including fish, living in the catchment areas of waterways and small and medium‐sized rivers in the surrounding areas where paddy field drainage flows in, rather than being kept in cages set up in specific paddy fields as in this study. This could have a greater impact.

In this study, various physiological changes observed in the appearance, visceral condition and blood properties of fish exposed to pesticide formulations containing THM, DT, and FN in the laboratory were similar to those observed in fish in paddy fields sprayed with pesticide formulations containing THM. There are many reports (Rania & Zaidi, 2023; Vignet et al., 2019; Windy et al., 2020; Weili et al., 2015) that animals exposed to NEOs have been shown symptoms such as decreased neutrophils, elevated antioxidant enzymes, increased lipid levels and ALT and ALP activity. Similar reactions have been observed in animals exposed to fipronil (Dallarés et al., 2020; Deiú et al., 2021; Kakuta, 2004). However, the concentrations of pesticide components in the water to which the fish were exposed in this study were determined taking into account the pesticide concentrations in the fish habitat, which were much lower than previously reported (THM; 1 and 20 μg/L, DT; 1.2 and 23.5 μg/L, FN; 0.01 and 0.2 μg/L). Thus, the biological effects of pesticide exposure may disappear after a short period of time, or superficially the appearance and disappearance of effects may be repeated, or the appearance period of effects may not be identical. Nevertheless, most biological effects of THM, DT and FN on fish found in the present study were similar. In other words, it was found that the effects of these pesticides, even at concentrations that can be detected in natural waters, are not significantly different from those already reported, that is, the direction of the effects on organisms does not change, at least during the exposure.

However, exposure to NEOs formulations containing THM and DT caused a decrease in plasma calcium concentration in the early stages (on week 1, the paddy field exposure and the exposure at a high concentration in the indoor experiment for THM, and 2 weeks after the start of the indoor exposure at a low concentration for DF), and the degree of scale detachment increased with prolonged exposure period. Exposure to FN‐containing pesticide formulations did not show such changes. In addition, these changes did not differ according to the degree of sexual maturity or sex. Therefore, it seems likely that the abnormalities in the body calcium balance suggested by reduced plasma calcium concentrations and subsequent scale detachment are among the specific reactions that appear early in exposure to NEOs.

It is reported that acetamiprid, imidacloprid, and nicotine have exert excitatory effects on nicotinic acetylcholine receptors in neonatal rat cerebellar neurons at concentrations above 1 μM (Kimura‐Kuroda et al., 2012). This means that NEOs may have adverse effects on human health, particularly on the developing brain. It has been reported that THM treatment to rat lead an alteration on the cholinergic transmission, inducing an increase in the anxiety behavior, decreasing in both acetylcholinesterase activity and the high‐affinity choline uptake (HACU) in synaptosomes from the hippocampus (Rodrigues et al., 2010). In another test, it was found that male mice administered clothianidin even if at the no‐observed‐adverse‐effect level (NOAEL) elevated in anxiety response during the plus‐maze test (Hirano et al., 2018).

A similar phenomenon has been reported in fish. The locomotor activity, such as enhanced aggregation and social activity, and increased abnormal swimming behavior, of adult zebrafish exposed to low concentrations (0.1 and 10 μg/L) of THM for 45 days was increased (Yang et al., 2023). Individual exposure to 1 mg/L nicotine for 3 min prevented conspecific alarm substance (CAS)‐induced anxiogenic‐like behaviors such as bottom‐dwelling, freezing, erratic movements in zebrafish (Duarte et al., 2019). Furthermore, it was found that exposure to NEOs at levels detected in environmental water was extremely short‐term, and that the effects persisted even after the water was returned to clean water (Takase & Kakuta, 2024). When Japanese medaka were exposed to THM (10 and 200 μg/L), DT (6 and 120 μg/L), and FN (1 and 20 μg /L) for 24 h, the fish were unable to exhibit normal anxiety behavior, that is, stay longer in the dark area. Furthermore, individuals exposed to the NEOs THM and DT were returned to clean water 1 week after exposure, and were shown to retain their abnormal behavior even after 2 weeks of rearing in clean water. Particularly, individuals exposed to THM and DT were returned to clean water 1 week after exposure, and were shown to retain their abnormal behavior even after 2 weeks of rearing in clean water. That is, the exposure to NEOs induce functional impairment in various parts of the fish body, including brain function, even at low concentrations and for extremely short periods, as well as even one‐time exposures, and the serious adverse effects may remain for long‐term.

Furthermore, this study investigated the spontaneous swimming and foraging behavior of fish reared in clean freshwater and after short‐term exposure to low concentrations of THM. There are many reports that the use of green and blue light is useful for promoting growth, improving survival rates, and reducing stress for many fish (Shibata & Arakawa, 2020), including flounder, sea bass and yellowtail. It has been reported that rockfish (Sakamoto et al., 2017) and flathead flounder (Ueki et al., 2019) respond well to blue color, and fish behavior becomes more active. It has been reported that when first feeding is used as an indicator for goldfish, green is good and red is bad (Song et al., 2016). On the other hand, it has also been reported that improper use of light can cause abnormalities in the shape of fish, poor feeding, and decreased survival rates (Shibata & Arakawa, 2020). However, this study found that if the fish were kept in a good environment, there was no difference in their behavior.

Additionally, the effects of THM exposure increases when fish are exposed to rapid changes in the external environment, such as forced transport or changing light conditions. It can be seen that fish subjected to THM treatment are strongly affected, become to more stimulate and maybe consume more oxygen. As a result, the exposed fish are more likely to have increased energy expenditure, less energy available for growth and reproduction, and reduced various physiological functions, including decreased immune function. In other words, even if the neonicotinoid insecticide THM is present at low concentrations and for a short period of time, fish exposed to it will not be able to lead a normal life unless they are in a stable environment with few external stimuli.

Finally, in bead feeding tests, fish exposed to THM increased the number of shiny white beads they put into their mouths under green LED illumination. The increased food requirements likely correspond to increased swimming activity and oxygen consumption rates of the fish. These results also indicate that fish exposed to low concentrations of THM for as little as a week remain affected in their feeding behavior, even if they are subsequently returned to fresh water. In addition, these fish showed markedly increased selectivity for shiny white beads under green LED light conditions, suggesting that flickering light can trigger feeding behavior in fish under certain light conditions. This indicates that there is possibility of a change in the ability of the fish's eyes to capture scattered light. Therefore, it suggests that the influence may extend not only to foraging behavior but also to a wide range of behaviors, including aggressive and migratory behaviors. The present study is the first point out that the exposure to THM at environmentally relevant concentrations, even for as short as a week, can negatively impact the survival of individuals or species by altering the foraging behavior of fish.

## AUTHOR CONTRIBUTIONS

All authors contributed to the study conception and design. All authors read and approved the final manuscript.

## CONFLICT OF INTEREST STATEMENT

There are no conflicts of interest to disclose in connection with this paper.

## ETHICS STATEMENT

This study was performed in a compliant manner with the Act on Welfare and Management of Animals (1973).
